# Chronic alcohol exposure, infection, extended circulating white blood cells differentiated by flow cytometry and neutrophil CD64 expression: a prospective, descriptive study of critically ill medical patients

**DOI:** 10.1186/2110-5820-2-50

**Published:** 2012-12-31

**Authors:** Arnaud Gacouin, Mikael Roussel, Antoine Gros, Elise Sauvadet, Fabrice Uhel, Loic Chimot, Sophie Marque, Christophe Camus, Thierry Fest, Yves Le Tulzo

**Affiliations:** 1 CHU Rennes, Maladies Infectieuses et Réanimation Médicale, Rennes, F-35033, France; 2 Inserm-CIC, Rennes, F-35033, France; 3 Univ Rennes 1, Faculté de Médecine, Biosit, Rennes, F-35033, France; 4 CHU Rennes, Laboratoire d’Hématologie, Rennes, F-35033, France; 5 Service des Maladies Infectieuses et Réanimation Médicale, Hôpital Pontchaillou, 2 rue Henri Le Guilloux, Cedex 9, Rennes, 35033, France

**Keywords:** Alcohol, At-risk drinking, Intensive care unit, Infection, Flow cytometry, CD64 cells

## Abstract

**Background:**

A history of prolonged and excessive consumption of alcohol increases the risk for infections. The goal of this study was to investigate circulating white blood cells (WBC) differentiated by flow cytometry and neutrophil CD64 expression in excessive alcohol drinkers versus abstinent or moderate drinkers, and in those with or without infection, in medical patients admitted to the intensive care unit (ICU).

**Methods:**

All patients admitted between September 2009 and March 2010 with an ICU-stay of 3 days or more were eligible for inclusion. Upon admission, hematological exams were conducted by flow cytometry.

**Results:**

Overall, 281 adult were included, with 37% identified as at-risk drinkers. The only significant difference found in circulating WBC between at-risk and not-at-risk drinkers was a lower number of B lymphocytes in at-risk drinkers (*P* = 0.002). Four groups of patients were defined: not-at-risk drinkers with no infection (n = 66); not-at-risk drinkers with infection (n = 112); at-risk drinkers with no infection (n = 53); and at-risk drinkers with infection (n = 50). Whilst the presence of infection significantly reduced levels of noncytotoxic and cytotoxic T lymphocytes and significantly increased levels of CD16^–^ monocytes in not-at-risk drinkers, with variation related to infection severity, infection had no effect on any of the variables assessed in at-risk drinkers. Post-hoc comparisons showed that B-lymphocyte, noncytotoxic, and cytotoxic T lymphocyte and CD16^–^ counts in at-risk drinkers were similar to those in not-at-risk drinkers with infection and significantly lower than those in not-at-risk drinkers without infection. Neutrophil CD64 index varied significantly between groups, with variations related to infection, not previous alcohol consumption.

**Conclusions:**

These results show that chronic alcohol exposure has an impact on the immune response to infection in critically ill medical patients. The absence of significant variations in circulating WBC seen in at-risk drinkers according to the severity of infection is suggestive of altered immune response.

## Background

A history of prolonged and excessive consumption of alcohol increases the risk for infections [1-3]. Not surprisingly alcoholism is a condition frequently encountered in patients admitted to the intensive care unit (ICU) with infection, especially those with pneumonia [4-8]. It is generally agreed that excessive alcohol use is associated with reduced host defenses and altered host repair but also that inflammatory response to infection differs depending on whether alcohol consumption is acute or chronic [9,10]. On one hand, alcohol impairs innate and adaptive immunity [10-13], whereas nonimmunologic factors potentially associated with chronic and excessive alcohol consumption, such as malnutrition, liver cirrhosis, poor dental hygiene, or active smoking, may contribute to the increased infection risk [2,3].

Immune alterations related to chronic alcohol exposure have been extensively studied *in vitro* and in animal models [10,12,14-18]. In humans, alterations in the immune system associated with chronic alcohol consumption have been described primarily in surgical patients [19-21]. In this group, alcohol abusers have shown a depressed CD4^+^ Th1 : Th2 ratio before and after surgery. In addition, the cytotoxic lymphocyte CD8^+^ : Tc1/Tc2 ratio was depressed preoperatively and remained depressed for 5 days. However, the impact of chronic alcohol consumption has not been as well described in critically ill medical patients [7,8,22,23].

The development of flow cytometry, its feasibility, and the increase in the number of cell surface-clustered domains identifiable by specific antibodies provides the opportunity to study alterations in the numbers of various circulating white blood cells (WBC) in large populations.

To further elucidate immune alterations associated with chronic alcohol exposure, we performed a study to assess differences between not-at-risk and at-risk drinkers with respect to circulating WBC and neutrophil CD64 expression in critically ill medical patients and the influence of coexisting infection on presentation to the ICU.

## Methods

### Patient enrollment

A prospective, observational cohort study was performed in the ICU at Hôpital Pontchaillou from September 15, 2010 to March 15, 2011. This ICU is a mixed 21-bed ICU admitting mostly medical patients in a 1,950-bed teaching hospital. In 2006, 31% of the patients admitted to this ICU were identified as at-risk drinkers, based on National Institute on Alcohol Abuse and Alcoholism (NIAAA) criteria [24,25]. Nonaplasic, medical, adult patients with an ICU stay of 3 days or more were eligible for the study if their admission was not due to acute alcohol consumption. We excluded pregnant women, patients declared to be deprived of their liberty by judicial or administrative decisions, patients who did not require blood sampling, and postoperative patients. The study was approved by the hospital’s Institutional Review Board. This noninterventional study did not require patient consent according to French law; however, information about the study was provided to the patient or their closest relative, who was informed that they had the option of refusing to contribute their samples or information to the study.

### Assessment of alcohol consumption

Assessments to determine alcohol consumption and categorization as at-risk or not-at-risk drinkers were similar to those used in a previous study [26]. Patients and/or their closest relatives were interviewed about medical history, dietary, and lifestyle habits. We systematically sought to determine the onset and duration of drinking and the average daily alcohol consumption. Whenever possible, information given by patients was confirmed by interviews with family members or family physicians.

### Definitions

At-risk and not-at-risk drinkers were classified according to criteria defined by the NIAAA. An at-risk drinker was defined as someone who had >14 drinks per week or more than 4 drinks per occasion for men aged ≤65 years, and as 7 drinks per week or more than 3 drinks per occasion for all women or men aged >65 years. Not-at-risk drinkers comprised abstainers (those who never drank alcohol) and moderate drinkers (2 or fewer drinks per day for men aged ≤65 years, and 1 drink or no drinks per day for all women or men aged >65 years) [25,27,28]. Patients with alcoholic cirrhosis were classified as not-at-risk drinkers when they had stopped their alcohol consumption 12 months or more before ICU admission.

Two intensivists and two specialists of infectious diseases retrospectively reviewed medical records and classified patients as not having systemic inflammatory response syndrome (SIRS) or sepsis, or as having SIRS, sepsis, severe sepsis, or septic shock at the time of admission to the ICU according to the consensus definitions [29]. Infection was considered as being hospital-acquired if it was diagnosed after 48 hours of hospital stay and was not incubating at admission.

Dental hygiene was grossly assessed by the same physician (AGa) for all patients and arbitrarily considered as “poor” when there was visual evidence of at least two untreated caries at examination. A tooth was classified as carious if there was evidence of cavity. Patients with body mass index <18.5 kg/m^2^ were defined as underweight [30].

### Data collection

Upon admission the following data were recorded: age, gender, body mass index, Simplified Acute Physiology Score II, Sepsis-related Organ Failure Assessment, presence of alcoholic liver cirrhosis, and, when available, serum levels of γ-glutamyl transferase (GGT), mean corpuscular volume (MCV), aspartate aminotransferase, and alanine aminotransferase. Current smoking also was considered.

In addition to the five types of circulating WBC classically differentiated by standard cytology (i.e., neutrophils, lymphocytes, monocytes, eosinophils, and basophils), we took the opportunity of routine flow cytometric evaluation (Hematoflow, Beckman Coulter, Miami, FL) using autogating software (Cytodiff CXP, Beckman Coulter) provided by the clinical hematology laboratory of our hospital to differentiate B lymphocytes, cytotoxic T lymphocytes, noncytotoxic T lymphocytes, natural killer lymphocytes, CD16-positive (CD16^+^) and CD16-negative (CD16^–^) monocytes, and immature granulocytes. Blood samples were performed at the time of ICU admission. Details of the flow cytometer used, data management, and routine application of flow cytometry have been published elsewhere [31]. The antibody combination used included fluorescein isothiocyanate conjugated CD36 (clone F16.152), phycoerythrin (PE), conjugated CD2 (clone 39C1.5), PE conjugated CRTH2 (clone BM16), PE-Texas Red conjugated CD16 (clone 3 G8), and PE-cyanine 7 conjugated CD45 (clone J.33). Twenty-eight healthy subjects served as a control group.

Also, because neutrophils play an important role as primary phagocytes, neutrophil CD64 expression, a diagnostic marker for infection and sepsis [32], was measured on the blood sample used for cytometry using a Leuko64 kit (Trillium Diagnostic, Brewer, ME) containing fluorescent beads, CD64, and CD163 antibodies analyzed with a FC500 flow cytometer.

### Study endpoints

The main study endpoint was to compare circulating subsets of WBC identified by flow cytometry and neutrophil CD64 indexes between at-risk drinkers and not-at-risk drinkers, whether they presented with infection at admission to the ICU or not. The secondary endpoint was to assess the influence of coexisting infection on subsets of WBC and neutrophil CD64 indexes in at-risk and not-at-risk drinkers.

### Statistical analysis

Proportions were compared by using the Chi-square test or Fisher’s exact test when required. Continuous variables were expressed as median values and interquartile ranges. The Mann-Whitney *U* test was used for comparisons between at-risk and not-at-risk drinkers. Four groups of patients were distinguished: not-at-risk drinkers with no infection; not-at-risk drinkers with infection; at-risk drinkers with no infection; and at-risk drinkers with infection.

Because the distributions of circulating WBC and neutrophil CD64 indexes were not normal in the four groups of patients or in the groups distinguished according to the severity of infection, the nonparametric Kruskal-Wallis test followed by Dunn’s multiple comparison post test were consequently used to evaluate the differences in circulating WBC counts and neutrophil CD64 indexes between patient groups in post-hoc analyses.

Forward multiple regression analyses were performed to determine whether at-risk drinking was an independent predictor of circulating B lymphocytes, noncytotoxic T lymphocytes, cytotoxic T lymphocytes, and CD16^–^ monocytes. In addition to infection and at-risk drinking, current smoking and poor dental state were entered into the model, because their proportions differed significantly between groups in the univariate analysis. Tests were two-sided, and *P* < 0.05 was considered to be statistically significant. For reasons of clarity, figures only show the subsets of WBC with significant variations after comparisons using the Kruskal-Wallis test.

## Results

### Patient characteristics

During the study period, 385 admissions had an ICU stay of 3 days or more. Sixty-three patients were not included in the study for the following reasons: 41 patients were admitted immediately after surgery (mainly liver transplantation); 3 patients were readmitted; 4 patients suffered from postchemotherapy aplasia; and 11 patients were admitted after acute alcohol consumption with a blood alcohol level >1 g/dL. In addition, nine patients were missed, and technical problems occurred in the laboratory for three patients. Among the 281 patients who were evaluated, 103 (37%) were classified as at-risk drinkers, of whom 3 patients (3%) had been at-risk drinkers for <5 years, 20 (19%) for 5–10 years, and 80 (78%) for >10 years. No patient was admitted with the diagnosis of acute alcoholic hepatitis.

### Comparisons between at-risk and not-at-risk drinkers

At-risk drinkers were predominantly males, were more frequently current smokers, and were more likely to have poor dental hygiene compared with not-at-risk drinkers (Table 1). Not-at-risk and at-risk drinkers also differed significantly for biomarkers of alcoholism. The only significant difference in circulating WBC between at-risk and not-at-risk drinkers was a lower number of B lymphocytes in at-risk drinkers (*P* = 0.002). To assess the effect of severe alcohol abuse in at-risk drinkers, we distinguished the patients with a daily intake of five or more drinks per day from those with a daily intake fewer than five drinks per day. The median number of circulating B lymphocytes was 0.117 × 10^9^/ L (0.03-0.14) in patients consuming ≥5 drinks per day and 0.145 × 10^9^/ L (0.08-0.18) in patients consuming <5 drinks par day (*P* = 0.04 after comparison).

**Table 1:** Baseline characteristics of patients and infections at admission to the intensive care unit

**Characteristics**	**Not-at-risk drinkers**	**At-risk drinkers**	***P *value**
	**N = 178**	**N = 103**	
General characteristics			
Age, median years (IQR)	58 (44–74)	61 (48–72)	0.64
Male, n (%)	101 (57)	78 (75)	0.001
SAPS II score, median points (IQR)	48 (33–60)	49 (35–59)	0.6
SOFA score, median points (IQR)	7 (4–10)	8 (4–11)	0.3
Biomarkers of alcoholism, median U/L (IQR)			
GGT	47 (24–101)	76 (36–232)	0.0001
MCV	91 (88–94)	96 (92–102)	<0.0001
AST (x 1 ULN)	1.29 (0.84–3.11)	2.14 (1.03–5.79)	0.03
ALT (x 1 ULN)	0.84 (0.46–1.77)	1.11 (0.63–2.26)	0.03
Prothrombin ratio (%)	74 (46–85)	71 (51–83)	0.88
Serum albumin (g/L)	30 (26–34)	29 (24–34)	0.58
Comorbidities, n (%)			
Current smoking	36 (20)	56 (54)	<0.0001
Underweight	20 (11)	11 (11)	0.88
Poor dental state	18 (10)	30 (29)	<0.0001
Alcoholic cirrhosis	12 (7)	13 (13)	0.1
Reason for admission, n (%)			0.4
Respiratory failure	63 (35)	41 (40)	
Central nervous system disorder	45 (25)	20 (19)	
Acute renal failure	10 (6)	4 (4)	
Cardiogenic shock	12 (7)	14 (14)	
Other	48 (27)	24 (23)	
Infection at admission, n (%)	112 (63)	50 (48)	0.01
Site of infection, n (%)			0.4
Pleural-pulmonary	66 (37)	33 (32)	
Central nervous system	14 (8)	2 (2)	
Urinary tract	9 (5)	5 (5)	
Other	23 (13)	10 (10)	
Etiology of infection, n (%)			0.35
Gram-negative bacilli	38 (21)	9 (18)	
Gram-positive cocci	41 (23)	20 (19)	
Virus	11 (6)	4 (4)	
Fungi	5 (3)	1 (1)	
Unknown	17 (9)	6 (6)	
Circulating WBC count, median x 10^9^/L (IQR)			
Neutrophils	11.45 (5.93–7.46)	9.94 (6.19–15.09)	0.44
B lymphocytes	0.19 (0.08–0.32)	0.13 (0.06–0.17)	0.002
Noncytotoxic T lymphocytes	0.59 (0.27–0.98)	0.49 (0.34–0.82)	0.6
Cytotoxic T lymphocytes	0.6 (0.3–0.12)	0.5 (0.3–0.1)	0.23
NK lymphocytes	0.4 (0.2–0.7)	0.4 (0.2–0.6)	0.38
CD16-negative monocytes	0.74 (0.4–1.14)	0.63 (0.32–1.21)	0.33
CD16-positive monocytes	0.09 (0.5–0.18)	0.11 (0.4–0.18)	0.92
Immature granulocytes	0.5 (0.2–0.24)	0.6 (0.2–0.19)	0.4
Neutrophil CD64 index >2, n (%)	70 (39)	35 (34)	0.38
Systemic inflammatory response, n (%)			0.1
No SIRS or sepsis	29 (16)	18 (18)	
SIRS	37 (21)	35 (34)	
Sepsis	59 (33)	28 (27)	
Severe sepsis or septic shock	53 (30)	22 (10)	

ALT, alanine aminotransferase; AST, aspartate aminotransferase; GGT, gamma-glutamyl transferase; IQR, interquartile ranges; MCV, mean corpuscular volume; NK, natural killer; SAPS II, Simplified Acute Physiology Score; SIRS, systemic inflammatory response syndrome; SOFA, sepsis-related organ failure assessment; ULN, upper limit of normal; WBC, white blood cells.

Sixty-five (58%) of the 112 not-at-risk drinkers with infection received antibiotics before admission to the ICU, whereas 35 (70%) of the 50 at-risk drinkers with infection received antibiotics (*P* = 0.15 after comparison). The median duration of symptoms was suggestive that infection before admission did not significantly differ between groups (3 days (range, 2–8) in not-at-risk drinkers versus 3 days (range, 2–5) in at-risk drinkers, *P* = 0.19). The two groups of patients were similar with respect to the site of infection the pathogens involved, and systemic inflammatory response (Table 1). Twenty at-risk drinkers (19%) and 33 not-at-risk drinkers (18%) died in the ICU.

### Effect of drinking status and infection

The influence of infection in not-at-risk and at-risk drinkers is documented in Table 2. B lymphocytes, noncytotoxic T lymphocytes, cytotoxic T lymphocytes, and CD16^–^ monocytes varied significantly when compared between the five groups of patients (see *P* values listed in the right most column). Post-hoc comparisons showed that for B lymphocytes, noncytotoxic and cytotoxic T lymphocytes, and CD16^–^ counts obtained for at-risk drinkers were similar to those in not-at-risk drinkers with infection and were significantly lower than those in not-at-risk drinkers with no infection (Table 2). Neutrophil CD64 indexes varied significantly between groups and clearly variations were related to infection and not to previous alcohol consumption. Indeed, proportions of patients with a neutrophil CD64 index >2 were lower than 15% in at-risk and not-at-risk drinkers without infection and approximately 55% in at-risk and not-at-risk drinkers with infection (Table 2).

**Table 2:** Comparison of comorbidities, subsets of circulating white blood cells, and neutrophil CD64 expression between not-at-risk drinkers with no infection, not-at-risk drinkers with infection, at-risk drinkers with no infection, and at-risk drinkers with infection

	**Control group**	**Not-at-risk drinkers with no infection**	**Not-at-risk drinkers with infection**	**At-risk drinkers with no infection**	**At-risk drinkers with infection**	***P *value**^†^
	**N = 28**	**N = 66**	**N = 112**	**N = 53**	**N = 50**	
Comorbidities, n (%)						
Underweight	-	7 (11)	14 (12)	6 (11)	4 (8)	0.87
Alcoholic cirrhosis	-	4 (6)	8 (7)	8 (15)	5 (10)	0.27
Current smoking	-	17 (26)	19 (17)	27 (56)	34 (68)	<0.0001
Poor dental state	-	7 (11)	11 (10)	15 (28)	15 (30)	0.0006
Antibiotics before admission, n (%)		5 (8)	61 (55)	5 (5)	30 (60)	<0.0001
Circulating white blood cells, x 10^9^/L (interquartile ranges)						
Neutrophils	3.8 (0.34-0.64)*	9.65 (6.78–12.93)	12.56 (5.15–17.73)	10.06 (5.71–15.31)	9.94 (6.83–14.47)	<0 .0001
B lymphocytes	0.43 (0.32-0.5)*	0.23 (0.88–0.41)**	0.16 (0.8–0.3)	0.12 (0.06–0.19)	0.15 (0.09–0.22)	<0 .0001
Noncytotoxic T lymphocytes	1.82 (1.62-2.27)*	0.71 (0.38–1.37)**	0.51 (0.21–0.85)	0.52 (0.38–0.78)	0.46 (0.27–1.42)	<0.0001
Cytotoxic T lymphocytes	0.3 (0.22-0.36)*	0.1 (0.05–0.18)**	0.05 (0.3–0.1)	0.06 (0.04–0.14)	0.05 (0.02–0.08)	<0 .0001
Natural killer lymphocytes	0.04 (0.03-0.05)	0.04 (0.02–0.08)*	0.04 (0.02–0.07)	0.04 (0.02–0.06)	0.04 (0.02–0.07)	0.75
CD16-negative monocytes	0.52 (0.44-0.56)*	0.86 (0.53–1.3)*	0.67 (0.34–1.33)	0.65 (0.43–1.11)	0.52 (0.22–1.13)	0.008
CD16-positive monocytes	0.06 (0.04-0.07)*	0.09 (0.05–0.16)	0.11 (0.04–0.21)	0.1 (0.06–0.18)	0.11 (0.03–0.16)	0.06
Immature granulocytes	0.08 (0.04-0.12)	0.05 (0.02–0.34)	0.05 (0.02–0.24)	0.06 (0.02–0.24)	0.08 (0.02–0.18)	0.80
Neutrophil CD64 index	-	1.02 (0.83–1.35)	2.25 (1.3–4.54)***	1.14 (0.93–1.55)	2.5 (1.09–4.43)***	<0.0001
Neutrophil CD64 index >2, n (%)	-	9 (14)	61 (54)	7 (13)	28 (56)	<0.0001

IQR, interquartile range.

^†^Global comparisons with Kruskal-Wallis test between the four groups of patients.

*P < 0.05 after *post-hoc* comparisons between control group and not-at-risk drinkers with no infection, not-at-risk drinkers with infection, at-risk drinkers with no infection and at-risk drinkers with infection.

**P < 0 .05 after *post-hoc* comparisons between not-at-risk drinkers with no infection and not-at-risk drinkers with infection, at-risk drinkers with no infection and at-risk drinkers with infection.

***P < 0 .05 after *post-hoc* comparisons with not-at-risk drinkers with no infection and at-risk drinkers with no infection.

At-risk drinking and infection were not found to be independent predictors of circulating B lymphocytes, cytotoxic T lymphocytes, and CD16^–^ monocytes after multiregression analysis. On the other hand, both at-risk drinking (β-coefficient = −0.174, standard error of β-coefficient = 0.07, *P* = 0.01) and infection (β-coefficient = −0.167, standard error of β-coefficient = 0.06, *P* = 0.01) were independently associated with noncytotoxic lymphocyte counts but not previous treatment with antibiotics (β-coefficient = −0.161, standard error of β-coefficient = 0.11, *P* = 0.17), current smoking (β-coefficient = −0.154, standard error of β-coefficient = 0.2, *P* = 0.24), and poor dental state (β-coefficient = −0.174, standard error of β-coefficient = 0.12, *P* = 0.15).

### Effect of infection severity

**Figure 1:**
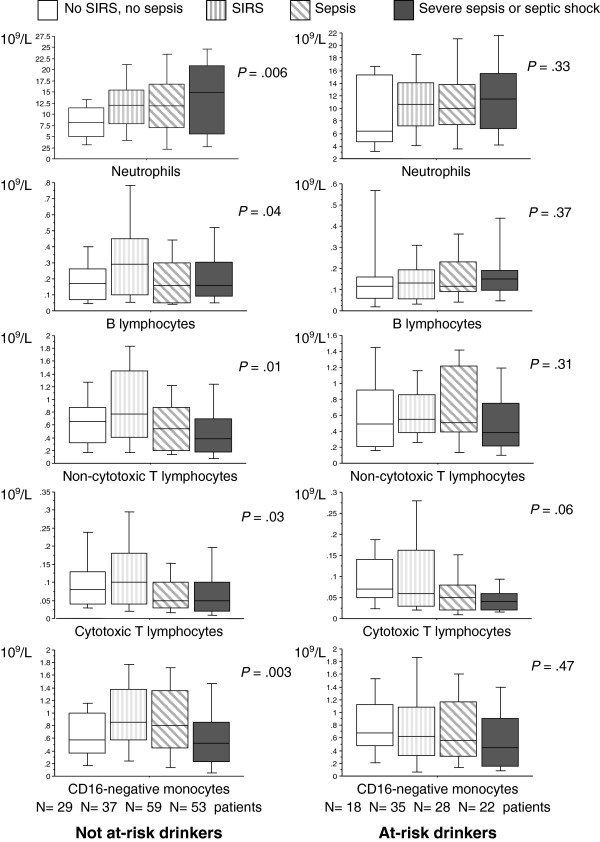
**Variation in counts of neutrophils, B lymphocytes, noncytotoxic T lymphocytes, cytotoxic T lymphocytes, and CD16-negative monocytes in not-at-risk and at-risk drinkers according to infection severity.** Data are presented as a box and whisker plot showing the median and the boundaries of the 25th and 75th percentiles, with the whiskers demonstrating the range. *P* values were obtained after comparisons by using the Kruskal-Wallis test. SIRS, systemic inflammatory response syndrome.

In the group of at-risk drinkers, none of the subsets of circulating WBC counts varied significantly after comparisons between patients with no SIRS or sepsis, patients with SIRS, patients with sepsis, and those with severe sepsis or septic shock (Figure 1), indicating that the severity of infection did not have an impact on WBC counts in at-risk drinkers. Conversely, in not-at-risk drinkers, neutrophils, B lymphocytes, and cytotoxic and noncytotoxic T lymphocytes (such as CD16^–^ and CD16^+^ monocytes) varied significantly according to infection severity.

**Figure 2:**
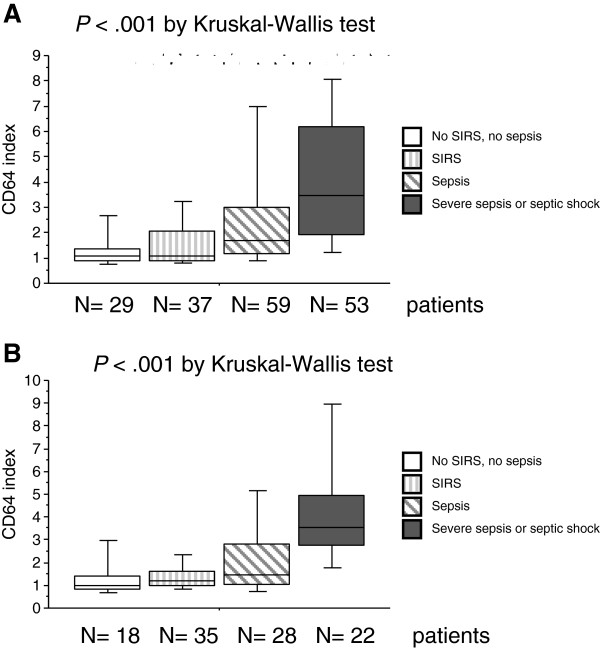
**Neutrophil CD64 index in (A) not-at-risk drinkers and (B) at-risk drinkers according to severity of infection.** Data are presented as a box and whisker plot showing the median and the boundaries of the 25th and 75th percentiles, with the whiskers demonstrating the range. SIRS, systemic inflammatory response syndrome.

Neutrophil CD64 index varied significantly by severity of infection in both at-risk and not-at-risk drinkers, being obviously higher in those with severe sepsis or septic shock in both patient groups (Figure 2).

## Discussion

This prospective, observational study performed on a large population of critically ill patients assessed upon admission to the ICU, and distinguished according to the presence or absence of infection, shows that previous alcohol consumption has an impact on counts of circulating WBC involved in both innate and adaptive immunity. The only difference in circulating WBC counts between at-risk and not-at-risk drinkers was in B lymphocytes, which were significantly lower in at-risk drinkers. We found that B lymphocytes, cytotoxic T lymphocytes, noncytotoxic T lymphocytes, and CD16^–^ monocytes in at-risk drinkers, with or without infection, were similar to those in not-at-risk drinkers with infection and significantly lower than those in not-at-risk drinkers without infection. When severity of infection was considered, none of the subsets of circulating WBC studied varied significantly in at-risk drinkers. Neutrophil CD64 index varied significantly by severity of infection both in at-risk and not-at-risk drinkers.

Because individuals with a history of alcohol abuse are more likely to develop severe pneumonia leading to ICU admission, the effect of chronic alcohol exposure on immunity of the lung has been assessed in many *in vitro* and *in vivo* studies [10,12]. In particular, it has been shown that ethanol consumption reduces neutrophil recruitment and neutrophil superoxide production during pulmonary bacterial infection and diminishes phagocytic activity, as well as cytokine and chemokine production by alveolar macrophages after lipopolysaccharide treatment [33,34]. Paradoxically, alterations in count and function of WBC associated with excessive chronic alcohol consumption have been less studied in the circulation than in the lung. To our knowledge, this is the first study performed in a large population of critically ill patients to assess the behavior of various subsets of circulating WBC in response to infection depending on whether or not patients were at-risk drinkers.

The low WBC counts in at-risk drinkers observed in our study are in accordance with published data showing that chronic alcohol exposure results in hyporesponsiveness of neutrophils to chemotactic signals, reduces delayed hypersensitivity response of isolated lymphocytes after stimulation *in vitro* by mitogens, and blunts CD4+ and CD8+ lymphocytes [2,10,12,14,35].

Various abnormalities in circulating neutrophils also have been described with chronic alcohol consumption, ranging from an increase in the number of these cells in the peripheral blood to neutropenia in those with the most severe form of infection or severe underlying hepatic disease [2]. For our part, we did not find that neutrophil counts differed significantly when compared between at-risk and not-at-risk drinkers at ICU admission. Interestingly, when functional activity of neutrophils was assessed by the expression of Fcγ receptor I, which is a marker of neutrophil activation recognized by the monoclonal antibodies CD64, we found that neutrophil CD64 expression varied significantly with infection and severity of infection, but not with alcohol consumption.

In the present study, most of the at-risk drinkers had been exposed to alcohol for many years. The low circulating B-lymphocyte count found in at-risk drinkers is in agreement with results from previous studies showing that the number of peripheral blood B cells is diminished after long-term alcohol consumption. Laslo et al. [36,37] previously showed that there is a decrease in the number of total B cells and the CD5+/CD19+ subset of B cells in patients with alcoholic liver cirrhosis and a decrease in number of CD5+ B cells in patients with active alcoholism that do not have liver disease. The number of circulating B cells also is reduced in mice undergoing chronic ethanol consumption [16]. Our results could be explained by a lower production of B lymphocytes in critically ill patients that were chronically exposed to alcohol and by alterations in the interactions between T and B lymphocytes. Previous experimental or clinical studies have shown a reduced number of cells in the thymus of patients chronically exposed to alcohol, a decreased activation of lymphocytes after antigen stimulation, reduced cytokine production by macrophages and T lymphocytes, and inhibited monocyte-derived myeloid cell capacity to induced T-cell activation [10,11].

Results listed in Table 2 are suggestive of an important impact of infection and chronic alcohol consumption on counts of circulating B lymphocytes, cytotoxic T lymphocytes, and CD16^–^ monocytes. However, for these subsets of WBC, multivariate analysis failed to demonstrate that at-risk drinking was an independent predictor when infection was included in the model. Because our findings suggest that at-risk drinkers admitted to the ICU with infection are less prompt to develop intense immune response than not-at-risk drinkers, we believe that systematic and accurate identification of patients with prior alcohol misuse will lead to improved care for these patients. In addition, our results suggest that neutrophil CD64 index may help physicians to diagnose infection in at-risk drinkers.

Whilst our study has some strengths, including a large number of patients and a long history of alcohol consumption in most of the at-risk drinkers, our study also has some limitations. The impact of alcohol exposure on the functionality of WBC was not assessed, except for neutrophils by looking at CD64 expression. Also, serum levels of immunoglobulins and cytokines were not analyzed in this study, whereas previous authors have shown that, at the onset of infection and during early septic shock, chronic alcoholic patients had lower plasma levels of proinflammatory interleukins than nonalcoholic patients [23]. Patients were assessed at different times during the course of infection, but it must be noted that at-risk and not-at risk drinkers did not differ in the duration of symptoms or in antibiotic therapy before admission.

Patients were not screened for illicit drugs and assessment of blood alcohol was not systematically performed; therefore, we cannot exclude that acute alcohol consumption was unrecognized in some patients. In human studies focusing on defects in the immune system associated with alcohol abuse, it is important to differentiate between acute and chronic alcohol exposure, and the presence or absence of acute hepatitis or liver cirrhosis. Acute alcohol consumption has effects on inflammatory cell activation opposite to those seen with chronic alcohol consumption [9,10]. In the present study, patients admitted with acute alcohol intoxication were excluded and very few patients had liver cirrhosis. Determination of at-risk drinking was based on results of interviews with patients and relatives regarding preadmission alcohol drinking habits. Therefore, some patients may have been misclassified, particularly due to underestimation of daily alcohol intake. However, the general characteristics of at-risk drinkers were similar to those previously reported by us [26] and others [38-40]. Even if biological tests have poor performance for screening at-risk drinking in critically ill patients, not-at-risk and at-risk drinkers differed significantly for liver enzymes, MCV, and GGT levels; thus, we believe that, in general, our patient groups were correctly classified. The proportion of patients with liver cirrhosis may have been underestimated. A liver biopsy should have been performed to eliminate or confirm with certainty the diagnosis of cirrhosis. Although not the main focus on the study, it is notable that as reported in a previous study [26] the proportion of patients with ICU-acquired infection was significantly higher in at-risk drinkers than in not-at-risk drinkers (data not shown).

## Conclusions

Our results show that, similar to findings in trauma and postoperative patients, chronic alcohol exposure has an impact on the immune response to infection in critically ill patients. In accordance with animal and experimental data, the absence of significant variations of circulating WBC seen in at-risk drinkers according to the severity of infection is suggestive of reduced immune response in patients chronically exposed to alcohol. On the other hand, neutrophil CD64 expression did not appear to be affected by chronic alcohol exposure.

### Key messages

– At-risk drinkers had a lower number of B lymphocytes at admission to the ICU.

– At-risk drinkers exhibited less variation in circulating WBC in response to infection than nonalcoholic patients.

– CD64 expression did not appear to be affected by chronic alcohol exposure.

## Competing interests

The authors declare that they have no competing interests.

## Authors’ contributions

AG, MR, AGr and YLT conceived the study protocol, participated in its designed and drafted the manuscript. MR, FU and TF carried out the cytometry. ES, LC and SM recorded data and helped draft the manuscript. AG and CC performed statistical analysis, CC helped interpret the data. All authors read and approved the final manuscript.
